# Social factors and Australian farmer suicide: a qualitative study

**DOI:** 10.1186/s12889-018-6287-7

**Published:** 2018-12-12

**Authors:** Meg Perceval, Victoria Ross, Kairi Kõlves, Prasuna Reddy, Diego De Leo

**Affiliations:** 1 National Centre of Excellence in Suicide Prevention, World Health Organization Collaborating Centre for Research and Training in Suicide Prevention, Australian Institute for Suicide Research and Prevention, Mt Gravatt campus, Griffith University, 176 Messines Ridge Road, Mt Gravatt, Brisbane, QLD 4122 Australia; 2 Faculty of Science, Engineering & Technology, Swinburne University of Technology, Melbourne, Australia; 3 Psychology and Neuroscience, Institute of Psychiatry, King’s College London, London, UK

**Keywords:** Farmer suicide, Stigma, Community attitudes, Help-seeking, Relationships, Social support, Rural communities, Biopsycho-ecological

## Abstract

**Background:**

Farmers and farm workers have been recognised as a group at high risk of suicide in Australia; however this risk is not without geographic and demographic variation. This study aims to identify and better understand the complex interplay of risk and protective factors surrounding farmer suicide, with an emphasis on social influences, so as to inform tailored and effective suicide prevention initiatives.

**Methods:**

Focus groups were conducted in three diverse sites across two states in Australia with men and women separately to gain perceptions about suicide risk and protective factors and attitudes towards suicide and help seeking. The three communities in each state represented areas with a suicide rate similar to, above, and below the state average. The communities were also diverse in their population, types of farming, geographic location, distance from and access to services. There were a total of 33 female and 30 male participants.

**Results:**

Qualitative analysis indicated three major interrelated social factors: (1) changing rural communities, (2) community attitudes and stigma and (3) relationship issues.

**Conclusions:**

The biopsycho-ecological model is considered useful to better understand and address social, as well as individual and environmental factors, pertaining to farmer suicide.

**Electronic supplementary material:**

The online version of this article (10.1186/s12889-018-6287-7) contains supplementary material, which is available to authorized users.

## Background

Risk of suicide in farmers has ‘been recognised to be higher than in other occupations in Australia and some countries across the world [1–7]. Whilst well documented, the phenomena of rural suicide remains poorly understood [8, 9]. Geographic and demographic variances in Australian farmer suicide cases suggest we need to look more carefully at the complex interaction of factors at play if targeted prevention strategies are to be developed and delivered to those most at risk [2, 10].

Research into the factors contributing to elevated rural suicide rates has found that factors such as the prevalence of mental illness did not explain discrepancies in rural compared to urban suicide rates, as rates of mental illness have not been found to be higher in rural areas [11, 12]. However, collective and contextual (or place) factors, such as socioeconomic decline in some rural areas, barriers to service utilisation, community attitudes towards mental illness and help-seeking, availability of firearms, and rural culture were found to be important as potential contributors to suicide [11].

Geographic and demographic variations in suicide rates have continued to be noted in Australian farmers [2, 13]. Taking person and place into account, and noting the importance of community and environmental contexts on well-being, have been cited as critical to any future rural suicide prevention intervention [14]. Farmers do not just belong to an occupational group, but are part of families, small and often tight-knit rural communities, and operate within a unique geographical, psychological, environmental and social context within Australia [15]. The farmers’ work environment has been described as characterised by “high stress” [16] with “relentless demands” [17]. Social factors such as relationship breakdown, isolation and loneliness, as well adherence to sociocultural norms of masculinity, stoicism and self-reliance have been implicated in Australian farmer suicide [10, 11, 18–21]. Farmers themselves perceive risk for suicide to result from a highly interactive combination of individual, environmental and social factors [20, 21].

Social and cultural context, alongside aspects of the physical environment and individual traits appear paramount when it comes to understanding and addressing farmer suicide. It is imperative we explore and better discern distinctive social influences to improve farmers’ health and wellbeing and reduce the toll of suicide in this vulnerable population. Given the limited existing research on relevant social factors in farmer suicide, the need for more qualitative studies examining these factors has been recognised [8, 10]. This qualitative study aims to identify and better understand the complex interplay of risk and protective factors surrounding farmer suicide, with an emphasis on social influences, so as to inform tailored and effective suicide prevention initiatives.

## Methods

The current study is part of a larger investigation into the perceived risk and protective factors and attitudes towards suicide and help seeking among farmers living and working on farms in two states in Australia: New South Wales (NSW) and Queensland (QLD). The study was approved by both Griffith (OTH/04/12/HREC) and Newcastle Universities’ (H-2013-0009) Human Research Ethics Committees, who partnered in the project, and was performed according to the Consolidated Criteria for Reporting Qualitative research (COREQ) checklist [22]. Elements of this study utilising the same data and methods have been reported on [20, 21].

Focus groups were conducted in three diverse sites across NSW and QLD to represent areas with a suicide rate equal to, above and below the state average. Communities were also diverse in terms of their geographic location, distance from services, population and agricultural enterprises. A purposive sampling framework was applied to recruit participants who lived and/or worked on a farm. Rural agencies with existing relationships to farmers in each site were sent out study information and invitations to participate via email. There were 30 male and 33 female participants (63) in total, across both states. Male and female focus groups were conducted separately as existing literature suggests that both men and women are more comfortable sharing their opinions openly in same-gender groups [23]. Participants received a $50 voucher as reimbursement for travel costs.

Across the three NSW sites, focus groups consisted of either six men or six women, with a total of 18 men and 18 women (36). Groups ranged from two to six participants in QLD and were supplemented with individual interviews due to difficulties with recruitment. In total there were 12 male and 15 female participants in QLD (27). Some QLD interviews took place via the telephone and others, along with the groups, in a hired venue. NSW groups took place in a venue provided by rural networks. Ages across both states and genders ranged from early 20s to mid-70s, with the majority of participants being aged between 35 and 65 years across all sites regardless of rurality. The inner regional site in QLD (average suicide rate compared to the state) engaged 6 male and 3 female participants, whereas both the remote (below average) and very remote (above average) sites engaged 3 male and 6 female participants. In NSW the suicide rates compared to state average in different ruralities were inner regional (below), outer regional (equivalent) and remote (above).

Informed written consent was obtained from all participants. The topic guide used for all groups and interviews comprised of 11 open-ended questions exploring farmers’ perceptions, knowledge and experiences of perceived suicide risk and protective factors, and attitudes towards suicide (see Additional file 1). NSW groups were facilitated by a female PhD candidate (MP) who worked in rural suicide prevention and lived and worked on a farm. This facilitator was known to some participants from one site in NSW. QLD groups were facilitated by a female registered psychologist with a postgraduate qualification and a female consultant with a PhD who were both unknown to participants. Follow-up access to mental health services was offered to all participants if required. Focus groups and interviews ran for between one and 3 h in QLD and approximately 3 h in NSW. All were audio recorded and professionally transcribed.

Methodological principles for social analysis as outlined by Krueger et al. (2001) [24] were followed when designing and conducting the study, with the social constructivism tradition guiding the research [25]. Focus groups were utilised in an attempt to understand how people who live and work of farms perceive and understand their environment and think and feel about suicide, rather than to try to build consensus or provide empirical reality [24]. Qualitative interpretation was conducted using NVivo software following the characteristics of qualitative research as described by Gibbs (2002) [26]. A generic qualitative approach [27] using inductive thematic analysis was undertaken, with coding and theme development directed by the content of the data [28]. Transcripts were read by two authors to generate initial codes and themes; three authors decided final themes and one author conducted comprehensive analysis. Validity of analysis was ensured via regular discussions with at least one other author to allow for reassessment of themes, continued interpretation and to gain consensus.

## Results

In the overall study, three interrelated and interdependent themes in terms of suicide risk factors emerged: individual, environmental and social. Individual [20] and environmental factors [21] have already been reported on. Here we focus and report on social factors. Under this theme, three major subthemes arose to explain both risk and protective factors: changing rural communities, community attitudes and stigma, and relationship issues.

### Changing rural communities

Participants across all groups described how they believed that quality of life in rural communities has decreased, as a result of declining rural populations and a reduction of services due to demographic and socioeconomic changes. Examples were provided of how some farming communities now comprised many less families and workers, and how people did not know their neighbours as well as previous generations once had. Participants spoke with nostalgia and regret about the decline in social gatherings such as weekend tennis matches between neighbouring families. People also described how in some areas there were now fewer venues available for community gatherings and that people generally had less time to organise and attend social get-togethers.

Participants described being busier and more time-poor for less financial gain compared to other times when farming was more economically viable. It was stated that fewer young people were returning to work on family farms on account of these pressures, and some participants stated they actively discouraged their children from returning to the farm. The need to undertake off-farm work for many male farmers due to financial hardship from drought and market pressures was highlighted, with resulting pressures on the women left behind to look after both the farm and the family.

The changing roles of women, with many women now also working off-farm, were evident. This was reflected in statements from older female participants about how difficult it was for younger families compared to “their day” when women traditionally remained on the farm to reduce domestic pressures and help bind families together. Farms becoming increasingly more difficult and financially unviable for families to maintain, and having fewer farm workers now employed, as well as an increase in farm ownership by larger corporate companies, was described as impacting not only individuals but also the social fabric of rural communities more broadly.

The transformation of rural communities and resulting decrease in social cohesion and integration were described by participants as key risk factors for suicide. Social interaction was described as not only a protective factor for suicide, but important for maintaining an overall sense of wellbeing.

*M1: Yeah, well there’s been a lot of change in the rural scene. Things have got tougher.*

*F1: There was a bit more money in the bush in those days.*

M2*: Farmers back then were happier in their lives. They would have been cranky about cattle prices, or that the government did this or that. But they were more socially happy in their lives than they are now… I always say to my wife “I wish I was born when my grandfather was born, because it looked like he had a lot more fun than (we do) now”.*

### Community attitudes and stigma

Negative community attitudes and stigma towards mental health issues and suicide were cited by participants as an issue for farmers and those living in rural areas. Despite recent efforts within the suicide prevention and mental health spaces in rural areas, there were numerous examples given that clearly depicted stigma in community attitudes towards help-seeking for financial or personal problems, the use of support services, mental health issues and suicide. The fear of having others in the community view or talk about them negatively and subsequently feeling like a failure was described as a compelling barrier to help seeking. Participants spoke of how people from small rural communities did not wish to be physically “seen” by others when attending certain services such as counselling or psychologists. This type of small town and community gossip, although generally negative, was also referred to by some in a positive way, in that people in the community were aware of what was happening to others, and could thereby support them.

Of note was the concept of self-stigma. A number of participants indicated that a person at risk of suicide might experience stigma through their own thoughts and perceptions far more than actually experiencing it through the words or the actions of others. A person’s self-judgement appeared to be a major limitation in terms of reaching out to others and as a primary cause of their suffering. Participants described this as intertwined with their sense of identity, fear of being a failure, and the normative “tough Aussie” culture; there was general agreement that these issues were more apparent for males. There appeared to be an underlying, ingrained assumption within the farming community and society at large that men in particular, but also rural women, should be able to cope without asking for help. Male participants admitted it would be difficult to have conversations about feeling depressed because of entrenched beliefs that help seeking was an indication of weakness.

Some individuals who had experienced mental health issues or strong adversity themselves, spoke about the great relief and healing power of being able to talk to someone about their problems. Participants described being able to talk to others openly about their problems, along with increased opportunities for social connectedness and interaction as outlined above, as protective factors against suicide risk.

F2: C*ertainly in farming communities and my experience in just this little community, is that it’s not okay to stand around at the pub and say “well I feel really terrible” or this or this… People can perceive they must be really suffering… but (1), it’s not okay to talk about it and (2), it is not okay to seek help.*

M3*: A sense of failure would prevent me from telling anybody (about personal problems).*

F3: *I think there’s a real attitude - well, my husband was raised by a generation who said “pull your socks up and get on with it”. So how on earth is he supposed to be equipped to have any other attitude than “I’ve got to be strong, it’s too shameful (to seek help)”.*

### Relationship issues

Participants described how, of the individuals they had known who had died by suicide, in many cases the person had experienced recent relationship difficulties or a separation/divorce from their partner, and this was particularly the case for male farmers. While relationship breakdowns were considered by a number of participants as a risk factor for suicide, having strong relationships with their partner and/or family members was as one of the main potentially protective factors against suicide. Several participants described how having a strong relationship with their partner and family support had helped them personally through times of difficulty. Lacking these relationships was described as contributing to a sense of social isolation and loneliness, which may exacerbate the inherent isolation of farming as an occupation and geographical isolation. The relationship between loneliness and maladaptive coping strategies such as increased alcohol intake was also raised in some of the groups.

Family tension on farms and in particular, generational succession was referred to as a source of stress, and in some cases, distress when family relationships broke down over disputes regarding succession agreements. The expectation felt by older and younger generations alike in passing on the farm; and the resulting pressure and discord brought to families was described by participants. Not being able to meet expectations to “carry on” the family farm was raised as another potential catalyst for males to feel a sense of failure and despair.

Participants indicated that social support did not necessarily need to come from relationships with a partner or family members: other relationships such as friendships and community interactions were also highlighted as important. Positive personal, neighbourly, community and even professional relationships were described not only has helping people to feel a sense of belonging, but also as giving meaning and purpose to life. Participants spoke positively of the role of workers or professionals in the community, the “accidental counsellors” who provided practical assistance (such as helping with paperwork) and social support. Strong relationships leading to social connectedness were repeatedly described as a panacea to many of the challenges experienced by farmers and as a key protective factor against suicide.

M4*: There’s been two (suicides) in my life, and they’ve both been relationship problems really. That was the kick over the edge… in both cases. So whether that is the only factor, I certainly know was the final factor.*

M5: *Lack of family support. If you are feeling isolated from your family and you have no one who really knows you in order to ask the right questions - “are you okay?” So if you are without that strong family member in your life to ask you a few prime questions it does make you feel isolated.*

M6: *That’s the whole thing about farming life, is it’s so unique in comparison to life in the city, and I’ve done both. You have extreme circumstances that you’re combating, every single day of your life… whether it be through drought, floods, succession, family partnership issues. We’ve had terrible problems within our family.*

## Discussion

Social factors have long been regarded important in determining both risk and protection for suicide [29, 30]. This study reported on farmers’ own perceptions of the relevant social factors to Australian farmer suicide. Three key subthemes were identified: changing rural communities, community attitudes and stigma, and relationship issues. These were described as being critical in protecting against, or increasing, suicide risk in farmers.

Agriculture is a large part of the Australian social identity; however, it has changed considerably over the years as the sector has been significantly challenged by environmental, sociodemographic and economic pressures [10]. These changes have influenced the social fabric of farming communities. Participants spoke of declining rural and farming populations, financial pressures due to environmental crises such as severe and protracted drought, and traditional sociocultural norms and gender roles changing, with women increasingly gaining off-farm work. These findings reflect available agricultural and national census data and social research [10, 18, 31, 32]. Increasing opportunities for social engagement and interaction were described by participants as a protective factor for suicide and to increase overall wellbeing for farmers. These findings are consistent with research suggesting that living in a declining area may impact negatively on mental well-being and that the combined constructs of community participation and personal social cohesion are strongly associated with all aspects of health, but most notably, mental health [33].

Our participants described perceived community attitudes and stigma as affecting both self-judgment and the likelihood of seeking help. This supports research by Fennell et al. (2018) [34] who found that stigma and subsequent reduced help seeking had more of an impact for mental, rather than physical health concerns in both rural men and women. Traditional gender norms and concepts of stoic masculinity have been found to be particularly evident in male dominated occupations and in the farming community [8, 35], and this was also reflected in our data, where gender differences were noted throughout focus group discussions. Where some participants described instances of how they overcame barriers such as stigma or gender norms to seek help, they spoke of this leading to positive outcomes. We concur with Fennell et al. (2018) [34] that a better understanding of barriers to seeking help in rural Australia is needed in order to develop methods to overcome these and facilitate help seeking behaviours.

Participants frequently referred to relationship problems, loneliness and social isolation as risk factors for suicide, with some cases of known suicides thought to result directly from relationship difficulties. Consistent with these accounts, Kunde et al. (2017) [19], in a psychological autopsy study, found relationship breakdown to be a key-precipitating factor in some farmer suicides. In our study, a lack of relationships and social support were described as being detrimental to health outcomes, and leading to maladaptive coping strategies such as increased alcohol use. Indeed, social isolation has been shown to be one of the strongest and most reliable predictors of suicidal behaviours across the lifespan [36].

Participants highlighted relationships as a key suicide protective factor and suggested that having strong relationships may assist in giving a sense of meaning and purpose in life. This is in keeping with previous research that suggested social support could be a useful protective factor against suicide [37], and other research [38] that found that the presence of meaning in life predicted decreased suicidal ideation over time and lowered lifetime odds of suicide attempt. A study of Australian farmers also concluded that increasing social support and a sense of belonging could improve mental health outcomes and act as a suicide protective factor in male farmers [39]. We suggest that strategies designed to both increase social connectedness and a sense of meaning in life may be particularly protective against suicide, as well as improving other health outcomes for farmers.

This research on social factors was a component of a larger study that also identified individual and environmental risk factors for suicide in Australian farmers [20, 21]. Given the complex interplay of social factors along with other individual and environmental risk and protective factors, a relevant model to assist in understanding and addressing farmer suicide is the expanded biopsycho-ecological model [40, 41], which acknowledges both the social and physical components of the environment in influencing health outcomes. Applying a biopsycho-ecological lens may be useful in developing much needed culturally appropriate and contextually sensitive suicide prevention strategies for farmers and farm workers.

### Study limitations

Recruitment issues in QLD meant that focus group size and format was not consistent across the two states. NSW groups consisted of six men and women respectively, and QLD groups/interviews involved between one and six participants, with some occurring by telephone. This was thought to be a combined result of the NSW facilitator having extensive rural and agricultural networks and living within a rural community, compared to the two different facilitators in QLD who did not and were both city-based, as well as distance and privacy issues that were put forth as barriers in QLD. Collection of data was staggered over time, with NSW groups occurring well before QLD groups. Despite the variations in recruitment, focus group format and the disparity between QLD and NSW farmer suicide rates [2], consistency of responses and congruency of themes was apparent across sites. It was anticipated that there may have been more variation given the differences of facilitators and group formats across the two states. Consistency of the interview schedule content and delivery is believed to have kept responses congruent. The NSW facilitator having extensive rural contacts clearly affected recruitment, participant numbers and group format (with QLD facilitators having to conduct some individual interviews over the phone), however it did not impact on the overall content of participant responses.

## Conclusion

This study provides important insights into the social risk factors for suicide as perceived by farmers themselves. As highlighted, suicide prevention programs that focus on increasing social interactions, reducing negative community attitudes and stigma towards mental health and help seeking, and increasing social support and connectedness, may provide protection against suicide and increase wellbeing for farmers. By addressing social factors, alongside individual and environmental considerations in rural communities we may improve farmers’ health and wellbeing and reduce the toll of suicide in this vulnerable population.

## Additional file

Additional file 1: Interview Schedule. This file contains the 11 open-ended questions that were asked across all focus groups and interviews. (DOCX 14 kb)

## Abbreviations

ARC: Australian Research Council
COREQ: Consolidated Criteria for Reporting Qualitative research checklist
NSW: New South Wales
QLD: Queensland
