# Supplementary material for: Social factors and Australian farmer suicide: a qualitative study
Source: BMC Public Health. 2018 Dec 12;18:1367. doi: 10.1186/s12889-018-6287-7 (PMC6292011; doi:10.1186/s12889-018-6287-7)
Supplement: Supplementary file 1 — Interview Schedule. This file contains the 11 open-ended questions that were asked across all focus groups and interviews. (DOCX 14 kb) [file 12889_2018_6287_MOESM1_ESM.docx]

**Interview Schedule**

**Questions**

**Risk factors**

1. What do you think influences or contributes to individual risk factors of suicide?
2. In your experience of people dying by suicide (known or heard of) are there any similarities you’ve noticed between the cases? What about differences?
3. What about communities - what may make certain communities more at risk of suicide?

**Protective factors**

1. What do you think could help someone who may experience suicidal thoughts?
2. What sorts of things have you heard of or have you experienced that may help protect an individual or community from suicide?
3. What do you think helps certain communities be more resilient than others?
4. What techniques or services or other types of help have you used, or others you know have used, when times have been tough, to help you pull through?

**Help seeking**

1. Do you think you could recognise suicidal thoughts and behaviours if you or someone around you was thinking about suicide?
2. What increases (or encourages) people to seek help if they are having problems and/or thinking about suicide?
3. What makes it difficult for people to seek help in your community?
4. How could we improve people’s knowledge of what services are available in their communities, and access to them? Do you think there are enough supports in your community, and are the supports that are available right for what people need?
